# Roup or Canker: Cause and Treatment

**Published:** 1898-07

**Authors:** H. A. Stevenson

**Affiliations:** Member of the Active Staff of London General Hospital; Demonstrator of Pathology, Medical Department Western University, London, Ont.


### ROUP OR CANKER: CAUSE AND TREATMENT.

By H. A. Stevenson, M.D., C.M.,
MEMBER OF THE ACTIVE STAFF OF LONDON GENERAL HOSPITAL; DEMONSTRATOR OF PATHOLOGY, MEDICAL DEPARTMENT WESTERN UNIVERSITY, LONDON, ONT.

Having always been interested in the rearing of chickens, and latterly, since 1891, in company with Dr. Niven, of this city, in the rearing of pheasants, my attention was often drawn to certain of the birds which seemed to have caught cold; afterward their wings drooped, and they invariably died. Treatment seemed of little avail.

In rearing young pheasants some of the young birds, after they had reached a certain age, would first attract attention by their droopy wings, death following soon after. Others shortly became affected in the same way, and died off rapidly, the total loss usually being considerable; sometimes half the flock would die off. On examining the mouths, a small whitish patch could be seen under the tongue, and by looking carefully similar small pinpoint patches would be visible on the roof of the mouth. These patches are pathognomonic of roup or canker.

*Symptoms.* The first symptoms of roup are not manifest for several days after the bird has been infected by the germ which causes the disease. On examining the mouth of the chicken which had been infected a few minute patches not larger than a pin's point may be seen, and the temperature, if taken per rectum, generally shows a rise of ½ to 1°. The temperature may be as high as 107°, and the hen going around apparently well and feeding well meantime. In a few days it gets mopish, and the eyes may swell, and, shaking its head, it gives a sound like "pit." In some cases it may appear to affect the eye only, the lids become œdematous and closed, and the eye may be even destroyed. Last month Dr. Niven had to remove the eye of an infected bird. The eye had been destroyed by the growth of the membrane, but after removal the bird recovered. In some cases the membrane creeps down the trachea or up into the nose, and from there it may affect the eye.

Roup is the cause of death in about 80 per cent. of young chickens that die. In taking up almost any poultry journal the reader is struck with the great number of roup specifics advertised. The diversity of these so-called specifics proves their worthlessness. I have tested a number of them. Such a variety of cures could not but fail in the treatment of a disease of such uniform character. It is a disease that presents the same clinical picture in nearly all cases. The symptoms, in the main, are the same, though some may have the foul-smelling discharge and others may be without it. Even poultrymen differ as to what roup is, and in asking poultrymen to bring to me chickens affected with roup, some brought in those without the offensive, foul-smelling discharge as roup, while others brought those with the offensive discharge as canker, and some said that they had some cases of roup which had become canker because the offensive discharge had appeared. Some birds affected with roup have a very disagreeable odor, but this odor has nothing to do with the disease, for the germ that causes this odor will produce the same odor when cultivated on blood-serum tubes, and the odor soon penetrates the whole incubator. This germ has nothing to do with the recovery of the bird, but it is generally found in most acute cases, and the bird recovers more quickly under treatment, probably because this discharge draws attention to their condition sooner.

*Temperature.* As to temperature, I think 105° is the normal temperature for a fowl. Chickens affected with roup have a temperature of 107° to 108° generally, but the temperature may run anywhere between 105° and 108.2°. There are other variations in temperature in healthy hens, which I have thought it is not necessary to mention.

*Sequelæ.* Paralysis. In quite a few cases paralysis result. Many chickens which have recovered from roup seem to be affected with a snuffling from the nose similar to that present during the attack; but this is not due to the disease being not cured, but to a paralysis of the palate muscles, and this effect will pass off in time.

*Leg-weakness in Fowls.* This is another form of paralysis which sometimes occurs. In 1892 I had a black Minorca cock that was troubled with what is called "leg-weakness," and finally it became so paralyzed that it could not stand, and died from starvation, not being able to get sufficient food. The postmortem showed a neuritis of the nerves supplying the legs. Since then one other case, which is described below, died in the same way. I have seen leg-weakness in other coops from the same cause. I think that nearly all cases of leg-weakness are due to roup where it is not due to injury.

*Bacteriology.* I believe roup in chickens to be similar to diphtheria in man, and roup to be caused by a specific germ which appears to me to be identical with the Klebs-Löffler bacillus. There is present in all cases of roup or canker this same germ. There are also, as is to be expected, several other varieties of germs present. Streptococci do not seem to be a very common associate, several forms of other cocci (staph.) and several forms of rod-shaped bacilli are present.

*Contagion.* As to infection from one fowl to another, it is a very communicable disease, even after the birds have been apparently cured. It may be conveyed by the drinking-dishes and feeding-troughs, as is shown in one experiment where I allowed one cock (mentioned above) to go without treatment for a time. The bird recovered with treatment, but developed leg-weakness. I then put him in with two rabbits and fed them all on grain from a small dish, so that their heads would rub together. The one rabbit developed the membrane in the eye, and died without treatment. The other developed the membrane in the nose and throat, and died. Cultures showed the same bacillus of roup; the postmortem showed the blood and organs sterile. No *coccidiæ* in either rabbit.

*Immunity to Roup or Canker.* To determine if the treatment by the serum given below would confer immunity, I put one cock infected with roup and one well hen together in a bag for about four hours, and then immunized the hen by injecting about 175 units; the hen escaped the disease. Then another cock, healthy, from another yard was put in the bag with the infected one and not immunized, and he developed the disease; then he was injected with the serum, 200 units, and he made a good recovery.

*Marketing of birds* which have been infected should be prohibited for three months after they have recovered, as they may easily spread the disease up to about this time. Birds suffering with roup should be removed from the rest of the flock and put in a coop by themselves where none of the rest of the birds can get near them, and separate drinking-dishes and feeding-pans used. After handling an infected bird the hands should be thoroughly washed with soap and water.

I believe roup and canker to be the same disease, a disease identical with diphtheria in man.

*Treatment.* No external treatment is of very much service. Some recommend brushing off the membrane and touching the part with nitrate of silver, but we have tried this in several dozen cases with no perceptible results and a very tedious recovery at the best in young pheasants. I would advise not to brush off the membrane, but to leave it to come off of itself. Burn any pieces of membrane that come off. The only treatment is by internal methods through the blood, by hypodermic injections of diphtheria antitoxic serum (Mulford's). This is the only treatment that is of any service.

This serum is made by growing the germ of diphtheria in beef-tea and then filtering out the germs, and using the filtrate which contains only the toxins; then injecting this in small quantities, at first, into a horse, then gradually increasing the doses until the horse can stand enormous doses. Then the horse is bled, and the clear serum that collects at the top of the coagulated blood is injected into the animal suffering with diphtheria or roup. If the serum is used early enough the animal will recover.

This antitoxin treatment we have carried out since the serum first came into use, and with excellent results. Antitoxin is non-poisonous, and too much given is better than too little. I give 150 to 225 units for a bird of about four to five pounds, a ten-pound bird about 250 units. This may be too large; if disease is taken in time, 50 to 100 units is sufficient. Generally one injection is all that is required. If necessary, 100 units may be given in two days afterward, but this is seldom needed. Since January 1st I have given about 150 injections, and since using the serum I have not lost one bird from roup, every bird recovering, though some would have a slight touch of paralysis for a time. The longer the bird is left without treatment the more chance there is of the bird dying, or paralysis is more likely to follow. The bird will recover if the serum is used. Two weeks ago I gave two birds an injection; one had a temperature of 108°, the other 107°; in three days after the injection one of them, the cock (107°), was crowing, and they both made a good recovery; the owner thought they were going to die and intended to kill them the same day that I got them; he was afraid they would infect his other birds. The serum that I have been using is that put up by the H. K. Mulford Company, of Philadelphia, called diphtheria antitoxic serum.

*The method of injecting* the serum is simple; any hypodermic syringe will do, but I prefer the syringe that the Mulford Company sells, because there is very little chance of it getting out of order, and also on account of the small piece of rubber-tubing that connects the needle with the barrel, so that if the bird wiggles around the needle will not be broken. When using an ordinary syringe give 10 to 17 minims of the serum if the serum contain 200 units per cubic cent.

*Mode of Injecting.* 1. Boil the syringe for three minutes in water before using it, and do not touch the needle afterward either with the hands or anything else, as it would be then infected and must be boiled again, or it may produce an abscess, something that has not happened to me in a single case.

2. Fill the syringe to the desired amount with the antitoxin serum (too much is better than too little), then see that all the air is out of the needle by elevating the point of the needle and pressing home the plunger till the serum escapes from the point.

3. Select any part where the tissues or skin are loose, under the wing is the best, or on the side between the ribs and the hip. Pinch up the skin between the fingers and introduce the needle well under the skin to nearly its full extent. It is not necessary to go into the muscle, the serum will be absorbed fast enough from the subcutaneous tissue. Then introduce the serum slowly by pushing home the plunger. It is not necessary to wash the part where the injection is made with an antiseptic solution before or after the injection, but withdraw the needle rapidly. After using the syringe, wash it and the needle out with water before putting them away, as the serum will clog up the needle when it is boiled again.

The reason that I write this article at this time is that this and next month will be the hatching months, and roup will play sad havoc with some flocks unless the poultry-breeder is prepared for it in time. Any medical man will, I am sure, give the first injection, and he will be able to procure the serum from any druggist.

Before closing, I have to thank Dr. J. S. Niven, President of the Canadian Kennel Club, for chickens and pheasants supplied for the first experiments. The doctor is using the serum when needed among his flocks with good results, and will answer any inquiry made as to the serum-treatment. (Inclose postage.)

Mr. McNeil, the well-known poultry-breeder, has been using the serum with good results.

P. S.—Since writing the preceding paragraphs I visited, with Dr. Niven, a poultry-yard where the owner had only two birds left out of a flock of fifteen, and five out of another flock of eighteen. He thought that the birds had died from cold, but on picking up one of the remaining sick birds the described membrane was present under and along the sides of the tongue and on the roof of the mouth. We injected the remaining birds.

---

The Bureau of Animal Industry will be prepared to distribute the hog-cholera serum as soon as there is announced the usual summer and fall outbreaks in the States where the disease has been prevalent for many years.

Secretary Wilson believes the great facilities and wide range of work of the Department of Agriculture could be used for post-graduate studies and work of all young men graduates of agricultural colleges, and that the department could thus have a large number of available men in special training for positions in this department, where special work is fitting men constantly, who are taken by institutions of learning for instructors. It is thought the President favors the plan, and, as it is said no money will be required, the plan is considered feasible. Would it not have been a remarkable source of saving and economy had preparation and proper recognition through an army veterinary corps been available in the purchase of the more than two million dollars' worth of horses and mules during the past three months? The unnecessary losses by disease and death would have saved enough to maintain this service for the next ten years.

